# New Introductions of Enterovirus 71 Subgenogroup C4 Strains, France, 2012

**DOI:** 10.3201/eid2008.131858

**Published:** 2014-08

**Authors:** Isabelle Schuffenecker, Cécile Henquell, Audrey Mirand, Marianne Coste-Burel, Stéphanie Marque-Juillet, Delphine Desbois, Gisèle Lagathu, Laure Bornebusch, Jean-Luc Bailly, Bruno Lina

**Affiliations:** Hospices Civils de Lyon, Lyon, France (I. Schuffenecker, B. Lina);; Centre Hospitalier Universitaire de Clermont-Ferrand, Clermont-Ferrand, France (C. Henquell, A. Mirand);; Université d’Auvergne, Clermont-Ferrand (C. Henquell, A. Mirand, J.-L. Bailly);; Centre Hospitalier Universitaire de Nantes, Nantes, France (M. Coste-Burel);; Centre Hospitalier de Versailles, Le Chesnay, France (S. Marque-Juillet);; Centre Hospitalier de Rambouillet, Rambouillet, France (D. Desbois);; Centre Hospitalier Universitaire de Rennes, Rennes, France (G. Lagathu);; Centre Hospitalier de Grasse, Grasse, France (L. Bornebusch)

**Keywords:** enterovirus 71, enterovirus species A, subgenogroup C4, Picornaviridae, France, viruses, humans, neonatal fever, meningitis, meningoencephalitis, rhomboencephalitis, phylogenetic analyses, herpangina, hand, foot and mouth disease

## Abstract

In France during 2012, human enterovirus 71 (EV-A71) subgenogroup C4 strains were detected in 4 children hospitalized for neonatal fever or meningitis. Phylogenetic analysis showed novel and independent EV-A71 introductions, presumably from China, and suggested circulation of C4 strains throughout France. This observation emphasizes the need for monitoring EV-A71 infections in Europe.

Human enterovirus 71 (EV-A71) is a member of the enterovirus species A in the family *Picornaviridae*, genus *Enterovirus*. On the basis of the 1D gene sequences encoding the VP1 capsid protein (1D^VP1^), EV-A71 has been classified into 3 genogroups (A–C) and 12 subgenogroups (A, B0–B5, C1–C5) (*1*); in addition, 3 new genogroups (D–F) were recently identified (*2*–*4*). In children, EV-A71 mainly causes asymptomatic or benign infections, such as neonatal fever and hand-foot and mouth disease (HFMD); less frequently, EV-A71 causes neurologic complications, such as encephalitis and poliomyelitis-like paralysis (*1*).

In the Asia–Pacific region, EV-A71 has emerged as a major public health concern over the past 15 years. Large outbreaks have been reported, associated with the emergence of new genogroups and subgenogroups, high rates of illness, and fatal cases of encephalitis (*1*,*5*). The largest epidemic expansion of EV-A71 occurred in China, mainly caused by EV-A71 subgenogroup C4 (EV-A71 C4) strains (*5*,*6*). By contrast, epidemic activity is low in Europe, where only 4 outbreaks of EV-A71 infection have been reported over the past 40 years: Bulgaria (1975), Hungary (1978), and the Netherlands (1986, 2007) (*5*,*7*). Most of the cases of EV-A71 infection reported since 1986 have been caused by subgenogroup C1 and C2 strains (*7*–*9*). In 2004, EV-A71 C4 strains were rarely detected in France, Germany, and Austria (*8*–*11*), and no other EV-A71 C4 cases were reported in Europe until 2012, when we detected C4 strains in 4 hospitalized patients, suggesting that dissemination of the C4 strains was restricted during 2004–2011. We describe the clinical cases caused by the EV-A71 C4 strains detected in 2012 and address the origin of these newly detected viruses.

## The Study

In France, EV infections diagnosed in hospital settings have been voluntarily reported to the National Institute for Public Health by a network of hospital laboratories since 2000 (*9*). In 2012, a total of 2,088 EV infections were reported by the laboratory network. In addition, in 2010, a total of 158 community cases of HFMD and herpangina were reported through a sentinel surveillance system implemented in Clermont-Ferrand, France (*12*). As part of the national surveillance, 1,249 EV strains were analyzed by 6 laboratories in the EV network (including the 2 National Enterovirus Reference Center laboratories in Lyon and Clermont-Ferrand). Of the 1,249 EV strains, 1,105 (88.5%) were successfully genotyped. Most of the genotyped strains were detected in cerebrospinal fluid (CSF) samples from patients with neonatal fever, meningitis, or meningoencephalitis or in samples from patients with HFMD or herpangina. Of the 1,105 genotyped EV strains, 16 (1.4%) were EV-A71 strains. Among these 16 cases of EV-A71 infection, a fatal case of rhomboencephalitis was diagnosed in an adult who had been treated with rituximab (*13*). On the basis of the complete 1D gene sequences encoding the VP1 capsid protein (1D^VP1^), 12 of the 16 EV-A71 strains were assigned to subgenogroup C2, and 4 were assigned to subgenogroup C4.

We conducted a retrospective review of medical records for the 4 patients with EV-A71 C4 infection to document the patients’ ages at diagnosis, clinical symptoms, length of hospitalization, and laboratory findings. The EV-A71 C4 infections were detected throughout the year in 3 regions (Brittany, Ile de France, and Provence-Alpes-Côte d’Azur). Of the 4 patients, 3 (6, 17, and 21 days of age) had neonatal fever when medical care was sought, and 1 patient (4 years of age) had meningitis (Table). The 21-day-old infant had persisting irritability and was hospitalized for 6 days. No severe neurologic complications were observed, and all 4 children had a favorable outcome. Bacterial culture results for CSF, blood, and urine samples from all 4 children were negative, and molecular detection results for herpes simplex virus types 1 and 2 and varicella-zoster virus in CSF were also negative. For the 3 children with neonatal fever, reverse transcription PCR was positive for EV-A71 in CSF specimens. For the child with meningitis, reverse transcription PCR was negative for EV-A71 in the CSF specimen, but an EV-A71 strain was isolated from a throat swab specimen.

**Table:** Characteristics of 4 patients with enterovirus 71 subgenogroup C4 infections, France, 2012*

Patient no., hospital location†	Date admitted	Age at diagnosis	Clinical signs	Laboratory values for CSF			Enterovirus RT-PCR results, C_t_‡			Duration, d	
				Leukocytes/ mm^3^	Protein, g/L		CSF	NP		Hospital stay	Antimicrobial drug use
1, Nantes	Feb 12	6 d	Fever syndrome	<2	0.54		Pos, 36.0	Pos, 36.2		4.0	4.0
2, Versailles	Mar 12	17 d	Fever syndrome	53	0.52		Pos, 31.7	NA		2.5	2.5
3, Grasse	Jun 12	4.5 y	Fever syndrome, meningitis	83§	0.92§		Neg	Pos, 27.4		1.0	0
4, Rennes	Oct 12	21 d	Fever syndrome, rhinitis	234	0.77		Pos, 30.0	NA		6.0	3.0

*For all 4 children, molecular testing results were negative for the detection of herpes simplex virus types 1 and 2 and varicella-zoster virus in CSF. In addition, for patients 1 and 4, molecular testing results were also negative for the detection of human herpesvirus type 6, cytomegalovirus, and Epstein-Barr virus. Results were also negative for CSF bacterial cultures. CSF, cerebrospinal fluid sample; C_t_, cycle threshold; NA, not available; Neg, negative NP, nasopharyngeal sample; Pos, positive; RT-PCR, reverse transcription PCR. †Nantes (47°13′N; 1°33′W; Brittany); Versailles (48°48′N; 2°08′E; Ile de France); Grasse (43°40′N; 6°55′E; Provence-Alpes-Côte d’Azur); Rennes (48°06′N; 1°40′W; Brittany). ‡The upper Ct for positivity for each of the RT-PCR assays used was 45.0. An in-house enterovirus real-time RT-PCR was used to test samples from patient 1. A commercial RT-PCR assay produced by Cepheid and commercialized by Orgentec (GP-EV-040, Orgentec, Trappes, France) was used to test samples from patients 2 and 3. Another commercial RT-PCR assay, Dia-ENT-050 (Diagenode, Seraing, Belgium) was used to test samples from patient 4. §CSF sample was hemorrhagic (10,300 erythrocytes/mm^3^).

Phylogenetic analyses based on a Bayesian approach were performed with a set of 97 1D^VP1^ sequences (Figure; Technical Appendix). The chronogram clearly shows that the EV-A71 C4 strains detected in France in 2012 (Figure, red branches) and those detected in France, Austria, and Germany in 2004 (Figure, green branches) belong to 2 separate lineages. The 4 strains detected in 2012 in France clustered with EV-A71 C4 strains detected in China during 2008–2011. We estimated that the most recent common ancestor of this cluster (Figure, blue branches) emerged, presumably in China, during 2006 (95% highest posterior density interval 2005–2007). Of the 4 EV-A71 C4 strains detected within a 4-month period in geographically distant (≈1,100 km) French regions, 2 displayed direct clustering (posterior probability 0.999) but substantial variation (genetic distance of 0.018 nt substitution per site between the corresponding 1D^VP1^ sequences). The quick evolution of the 2 EV-A71 C4 strains from their common ancestor suggests local spread of the viruses. The other 2 strains displayed consistent clustering (posterior probability >0.8) with different strains isolated in China, suggesting independent introductions of virus.

**Figure:**
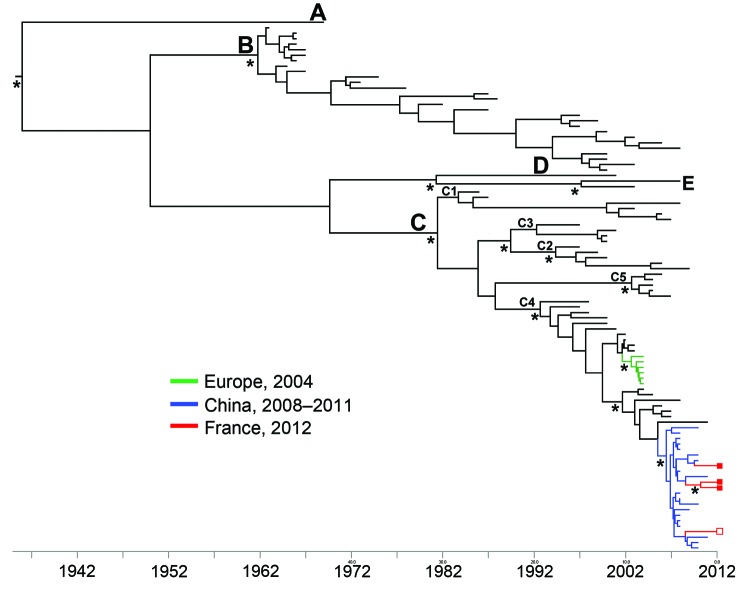
Dated phylogeny inferred by using 97 enterovirus 71 (EV-A71) 1D gene sequences encoding the VP1 capsid proteins (1D^VP1^). The dataset included the 4 sequences determined in this study; 37 sequences from EV-A71 C4 strains detected in Austria, China, Korea, France, Germany, Japan, and Taiwan during 1998–2011; and 57 sequences from prototype and clinical strains representative of the genogroups and subgenogroups A, B1–B5, C1–C5. The tree topology shows the relationships between the strains isolated in France during 2012 and the strains circulating in China. The *x*-axis represents sampling years. The phylogenetic relationships were inferred with complete 1D^VP1^ gene sequences (891 nt) by using a Bayesian method (BEAST software; http://beast.bio.ed.ac.uk). The tree was reconstructed using FigTree 1.4.0 (http://tree.bio.ed.ac.uk/software/figtree). Asterisks at key nodes indicate posterior probability values >0.99. Red indicates the 1D^VP1^ sequences determined in this study: complete sequences (GenBank accession nos.KF900159–61) are represented by closed squares on the right side of the figure; a partial sequence (298 nt) (GenBank accession no.KF900162) is represented by an open square). Green indicates the 1D^VP1^ sequences from EV-A71 C4 strains detected in France, Germany, and Austria in 2004. Blue indicates the 1D^VP1^ sequences from EV-A71 C4 strains detected in China during 2008–2011. Letters A–E indicate genogroups; C1–C4 indicate subgenogroups.

## Conclusions

In 2012, EV-A71 C4 strains were detected in France in 4 children hospitalized for neonatal fever or meningitis. Although EV-A71 C4 strains have circulated extensively in China since 2008, this virus has rarely been detected in Europe. In France, 133 cases of EV-A71 infections were reported during January 2000–May 2013 (*9*) (I. Schuffenecker, unpub. data). EV-A71 C2 infections have been predominant since 2007; however, only 5 cases of EV-A71 C4 infection have been identified in the country since 2004. Our Bayesian analyses excluded a direct evolution of the 2012 EV-A71 C4 strains from the earlier 2004 European virus lineage. The phylogenetic data are consistent with 3 independent virus introductions, presumably from China, and are compatible with a more global circulation of subgenogroup C4 enteroviruses. In 2013, the C4 subgenogroup also emerged in Russia, where it was associated with an outbreak of 78 reported cases, including 1 fatal case of meningoencephalitis (*14*).

Many cases of fatal encephalitis have been associated with EV-A71 C4 infection outbreaks in China (*6*), which highlights the neurovirulence of EV-A71 strains. Rare acute flaccid paralysis cases have also been reported in Australia through the national poliomyelitis surveillance program (*15*). Although the prevalence of neurologic cases associated with EV-A71 infection is currently low in Europe, the recent circulation of EV-A71 C4 in France and in Rostov, Russia (along the eastern border with Europe), underscores the need for improved surveillance of neurologic manifestations associated with EV infection and of the incidence of HFMD within communities. In addition, careful monitoring for the possible introduction and circulation of new EV-A71 genogroups and subgenogroups should be conducted.

Technical Appendix Dated phylogeny inferred by using 97 enterovirus 71 1D gene sequences encoding the VP1 capsid proteins; taxon names of the strains selected for the phylogenetic analyses are shown.
