# Supplementary material for: New Introductions of Enterovirus 71 Subgenogroup C4 Strains, France, 2012
Source: Emerg Infect Dis. 2014 Aug;20(8):1343–6. doi: 10.3201/eid2008.131858 (PMC4111202; doi:10.3201/eid2008.131858)
Supplement: Technical Appendix — Dated phylogeny inferred by using 97 enterovirus 71 1D gene sequences encoding the VP1 capsid proteins; taxon names of the strains selected for the phylogenetic analyses are shown. [file 13-1858-Techapp-s1.pdf]

Article DOI: http://dx.doi.org/10.3201/eid2008.131858

New Introductions of Enterovirus 71 Subgenogroup C4, France, 2012

Technical Appendix

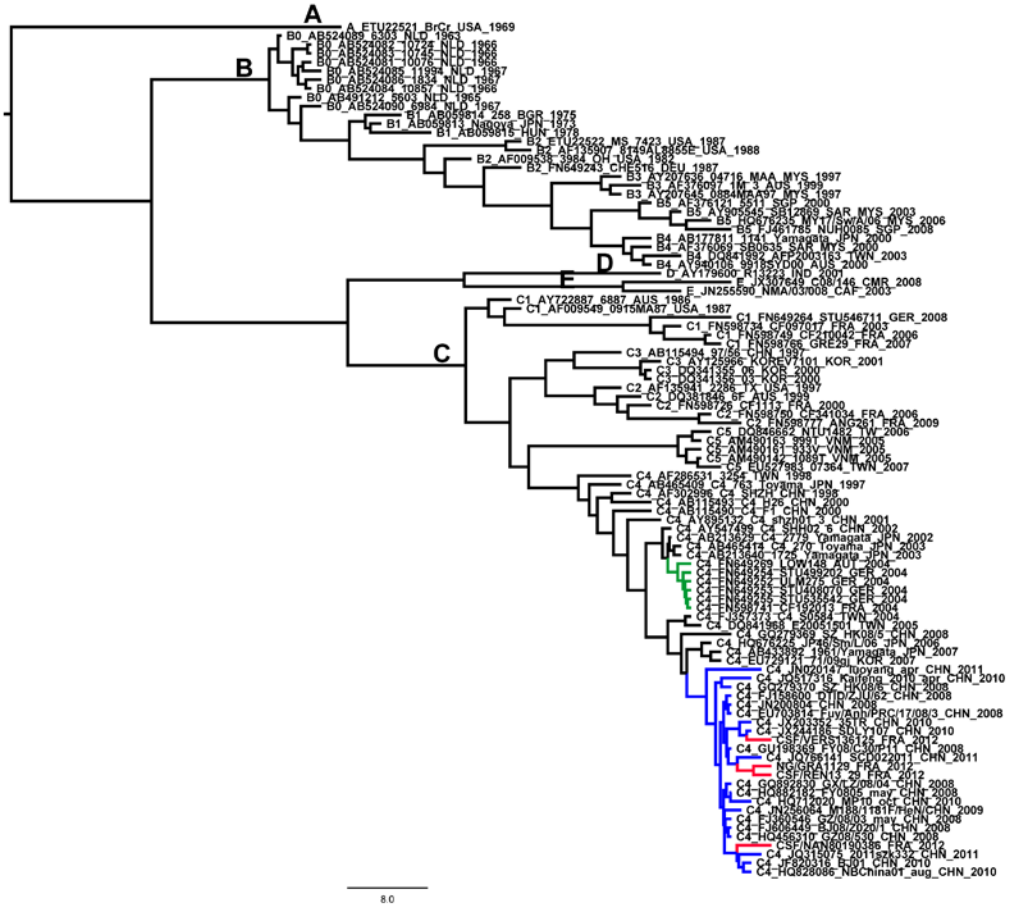

Technical Appendix Figure. Dated phylogeny inferred by using 97 enterovirus 71 (EV-A71) 1D gene sequences encoding the VP1 capsid proteins ($1D^{VP1}$). The figure shows the taxon names of the strains selected for our phylogenetic analyses. The dataset included the 4 sequences determined in this study; 37 sequences from EV-A71 C4 strains detected in Austria, China, Korea, France, Germany, Japan, and Taiwan during 1998–2011; and 57 sequences from prototype and clinical strains representative of the genogroups and subgenogroups A, B1–B5, C1–C5. The tree topology shows the relationships between the strains isolated in France during 2012 and the strains circulating in China. The phylogenetic relationships were inferred with complete $1D^{VP1}$ gene sequences (891 nt) by using a Bayesian method (BEAST software, http://beast.bio.ed.ac.uk). The tree was reconstructed by using FigTree 1.4.0

(http://tree.bio.ed.ac.uk/software/figtree). The length of horizontal branches was drawn to the indicated scale (nucleotide substitutions per site per year). Red indicates the $1D^{VP1}$ sequences determined in this study. Green indicates the $1D^{VP1}$ sequences from EV-A71 C4 strains detected in France, Germany, and Austria in 2004. Blue indicates the $1D^{VP1}$ sequences from EV-A71 C4 strains detected in China during 2008–2011.Letters A–E indicate genogroups; C1–C4 indicate subgenogroups.
